# Estimation of groundwater storage loss for the Indian Ganga Basin using multiple lines of evidence

**DOI:** 10.1038/s41598-023-28615-y

**Published:** 2023-01-31

**Authors:** Sreekanth Janardhanan, Akhilesh S. Nair, J. Indu, Dan Pagendam, G. S. Kaushika

**Affiliations:** 1 CSIRO Land and Water, Dutton Park, QLD Australia; 2 Geophysical Institute, University of Bergen and Bjerknes Centre for Climate Research, Bergen, Norway; 3 Indian Institute of Technology, Bombay, Powai, Mumbai, Maharashtra India; 4 Interdisciplinary Centre for Climate Studies, Indian Institute of Technology, Bombay, Powai, Mumbai, India; 5 CSIRO Data 61, Dutton Park, QLD Australia; 6 Indian Institute of Technology, Roorkee, India

**Keywords:** Hydrology, Civil engineering

## Abstract

We used remote sensing data, field observations and numerical groundwater modelling to investigate long-term groundwater storage losses in the regional aquifer of the Ganga Basin in India. This comprised trend analysis for groundwater level observations from 2851 monitoring bores, groundwater storage anomaly estimation using GRACE and Global Land Data Assimilation System (GLDAS) data sets and numerical modelling of long-term groundwater storage changes underpinned by over 50,000 groundwater level observations and uncertainty analysis. Three analyses based on different methods consistently informed that groundwater storage in the aquifer is declining at a significant rate. Groundwater level trend indicated storage loss in the range − 1.1 to − 3.3 cm year^−1^ (median − 2.6 cm year^−1^) while the modelling and GRACE storage anomaly methods indicated the storage loss in the range of − 2.1 to − 4.5 cm year^−1^ (median − 3.2 cm year^−1^) and − 1.0 to − 4.2 cm year^−1^ (median − 1.7 cm year^−1^) respectively. Probabilistic modelling analysis also indicated that the average groundwater storage is declining in all the major basin states, the highest declining trend being in the western states of Rajasthan, Haryana and Delhi. While smaller compared to the western states, average groundwater storage in states further towards east—Uttar Pradesh, Bihar and West Bengal within the basin are also declining. Time series of storage anomalies obtained from the three methods showed similar trends. Probabilistic storage analysis using the numerical model vetted by observed trend analysis and GRACE data provides the opportunity for predictive analysis of storage changes for future climate and other scenarios.

## Introduction

Aquifers of the Ganga Basin constitute the major groundwater storage in the northern Indian region and comprise one of the largest reservoirs of groundwater in the world. Recent studies^1–4^ that analysed satellite and regional groundwater monitoring data indicated that long-term changes in monsoon precipitation and groundwater abstraction are driving groundwater storage variability resulting in a declining trend in groundwater storage at a rate of 2 cm per year in the region. Another recent study^5^ shows that, the lowering of water level observed in several lower Indian reaches of the Ganga River, is related to reduction in groundwater base flow into the river. These findings demonstrate that, there is a strong inter-relatedness among biophysical systems across climate, surface water and groundwater hydrology and evolving water use for agriculture and other purposes in the basin.

Several studies in the recent past have analysed remote sensing datasets to investigate storage changes in the northern Indian region and elsewhere. Some of these studies^3,4,6–9^ used Gravity Recovery and Climate Experiment (GRACE) data, to investigate loss of groundwater storage. Ground-based observations are required to further constrain the coarse-scale storage change estimations from remote sensing data. MacDonald et al.^2^ used in-situ water level observations to quantify groundwater depletion and quality in the Indo-Gangetic Basin. While several studies have investigated long-term groundwater storage changes in the northern Indian regions and Indo-Gangetic Basin by the analyses of remote sensing and field observations, there is a dearth of studies that comprehensively investigated and accounted for the dynamics of groundwater flow in the regional aquifer system and its uncertainties considering aquifer storage and hydraulic characteristics. To the best of our knowledge no past groundwater modelling studies at the regional aquifer-scale have accounted for variabilities in groundwater storage characteristics in quantifying trends in groundwater storage changes in the Ganga Basin. While a regional numerical groundwater model was developed in the recent past for the Ganga Basin^10^, the study did not consider parameter uncertainties and its effect on the estimation of groundwater balance and changes in storage.

Assessment of dynamic storage changes in the regional aquifer warrants accounting of uncertainties in storage estimation caused by influencing properties like specific yield and hydraulic conductivity in the heterogeneous aquifer system. Development of a numerical groundwater balance model that can account for uncertainties in recharge, groundwater abstraction, and hydraulic characteristics can be used to probabilistically quantify the groundwater balance and trends in groundwater storage changes. Such analysis can be used in conjunction with the analyses of in-situ water levels and remote sensing data sets to conclusively assess long-term storage trends and its uncertainties. The remote sensing (GRACE) approach estimates groundwater storage loss as the difference between total Terrestrial Water Storage (TWS) and other vertical water balance components. Numerical modelling and uncertainty analysis at the regional aquifer scale provides the opportunity to more directly account for groundwater balance components using their posterior probability ranges underpinned by groundwater observation data. Thus, numerical modelling-based estimates can provide an independent estimation and verification of storage changes while accounting for simulation uncertainties contributed by aquifer hydraulic properties as well as uncertain water balance components including recharge and river-aquifer interaction. This study addresses the gap in such concerted efforts for integrating available data sets in a regional scale groundwater model that enables dynamic assessment of groundwater balance and storage changes in the regional aquifer system.

This study investigated trends in groundwater storage in the regional aquifer in the Ganga Basin and developed a numerical groundwater model for simulating the long-term average groundwater storage changes. Three parallel and complementary analyses were used comprising: (a) trend analysis of observed groundwater levels data for the period 1996 to 2017 from the regional monitoring network of CGWB using Mann Kendall and Theil Sen’s slope; (b) groundwater storage anomaly estimation using GRACE and Global Land Data Assimilation System (GLDAS) data sets; and (c) probabilistic numerical modelling and uncertainty analysis of the aquifer water balance considering heterogeneity in hydrological and hydrogeological parameters and forcing data. The GRACE data analysis and numerical modelling was undertaken for overlapping periods between Jan-2003 and Dec-2016. A probabilistic approach was used for numerical modelling by running 500 calibration constrained simulations of the numerical model, underpinned by 50,793 groundwater head observations from across the regional aquifer system.

**Figure 1:**
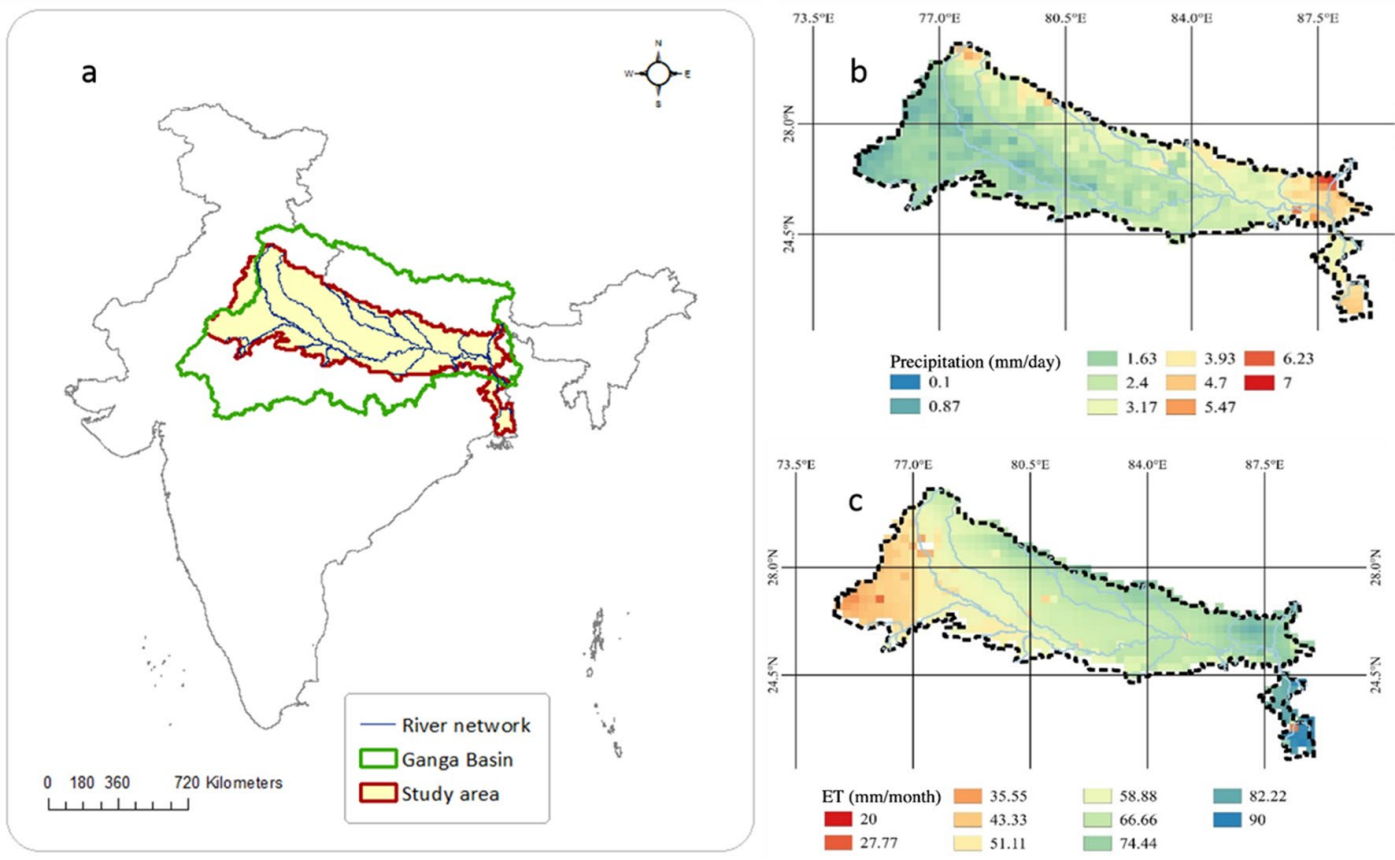
(**a**) Location of the study area within the Ganga Basin. (**b**) Mean precipitation during a period of 14 years from 2003 to 2016. (**c**) Mean evapotranspiration during a period of 14 years from 2003 to 2016.

**Figure 2:**
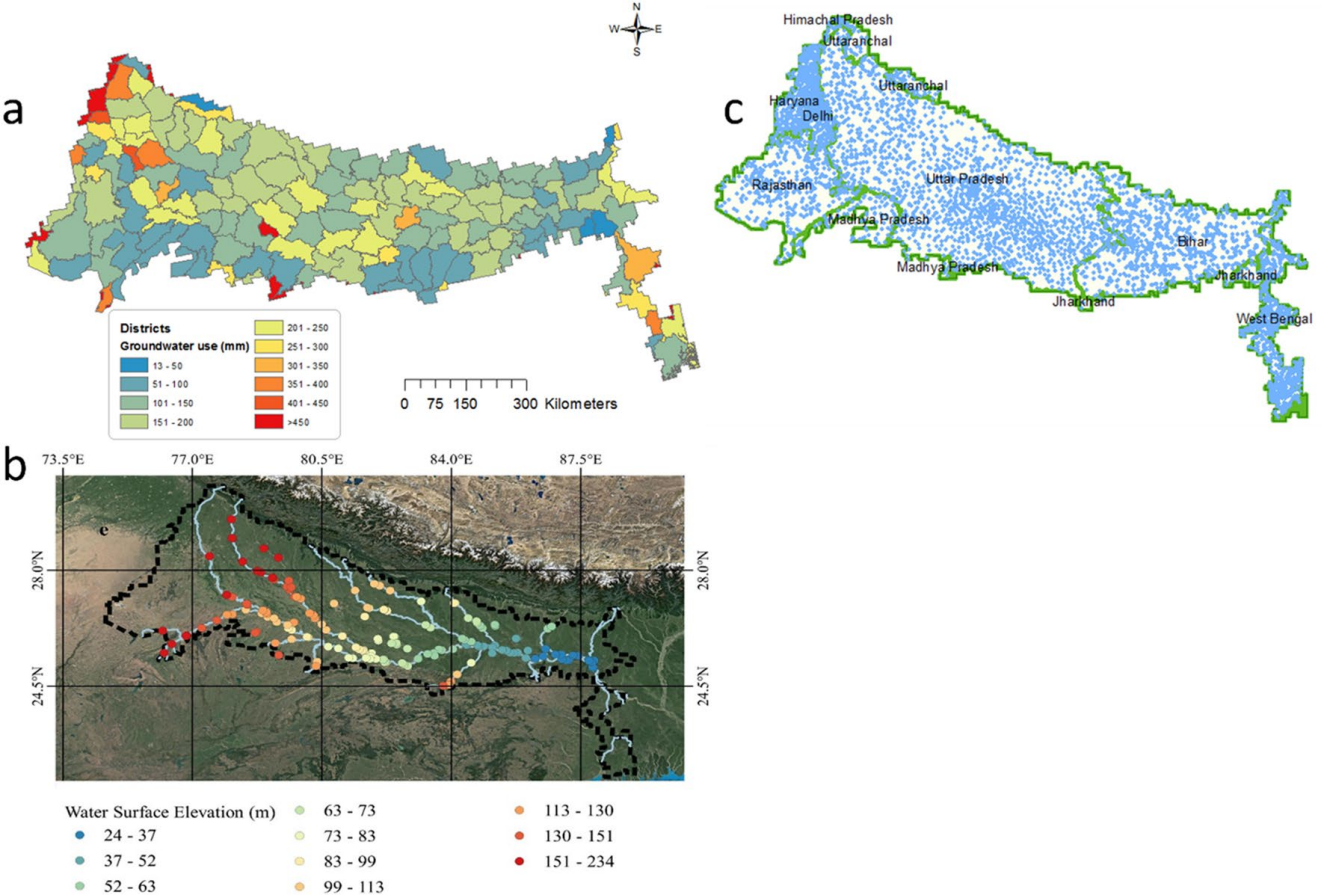
(**a**) Estimated annual groundwater abstraction at the district scale. (**b**) Mean water surface elevation during a period of 14 years from 2003 to 2016. (**c**) Location of monitoring bores and state boundaries within the model area.

The study area is shown in Fig. 1a. The data sets used in this study include the a) GRACE monthly mass grids—global mascons data between 2003 to 2016 of Center for Space Research (CSR) b) the groundwater level records between 1996 and 2017 of Central Groundwater Board c) estimated district scale annual groundwater extraction rates (CGWB, 2019, Fig. 2a) d) GLDAS Noah land surface model L4 monthly evapotranspiration estimates (Fig. 1c), e) In-situ gridded precipitation data from India Meteorological Department (Fig. 1b). In addition to this observations river stages information is estimated using satellite altimetry (Fig. 2b). The timeseries of altimeter products used in this study were specified by the Laboratory of Space Geophysical and Oceanographic Studies (LEGOS) and computed by Collecte Localisation Satellites (CLS) on behalf of Centre national d'études spatiales (CNES) and Copernicus Global Land. Figure 2b depicts the average water surface elevation in virtual stations in Ganges basin during a period of fourteen years from 2003 to 2016. Figure 2c shows the major states and locations of observation bores in the basin.

## Groundwater level trends

Groundwater levels are monitored in the Ganga Basin with the regional monitoring network established by the Central Groundwater Board of India. Water levels are monitored 4 times per year by this network. These four measurements correspond to one each in pre-monsoon (March–May), monsoon (June–September), Kharif (July–October) and Rabi (October to March) seasons respectively. Groundwater level monitoring data from a total of 5075 observation bore location within the boundaries of the study areas were chosen for the trend analysis. The data set was further filtered to select bores with a minimum of 20 observations within the chosen time period of Jan-1996 to Dec-2017 resulting in selection of 2851 bores for Mann–Kendall Tau and Theil Sen slope statistical analyses for groundwater level trends. Mann Kendall Tau statistic was used for evaluating the nature (increasing/decreasing) and strength of the trend. Sen’s slope provides the rate of increase or decrease of groundwater levels where consistent change is observed over a long period.

**Figure 3:**
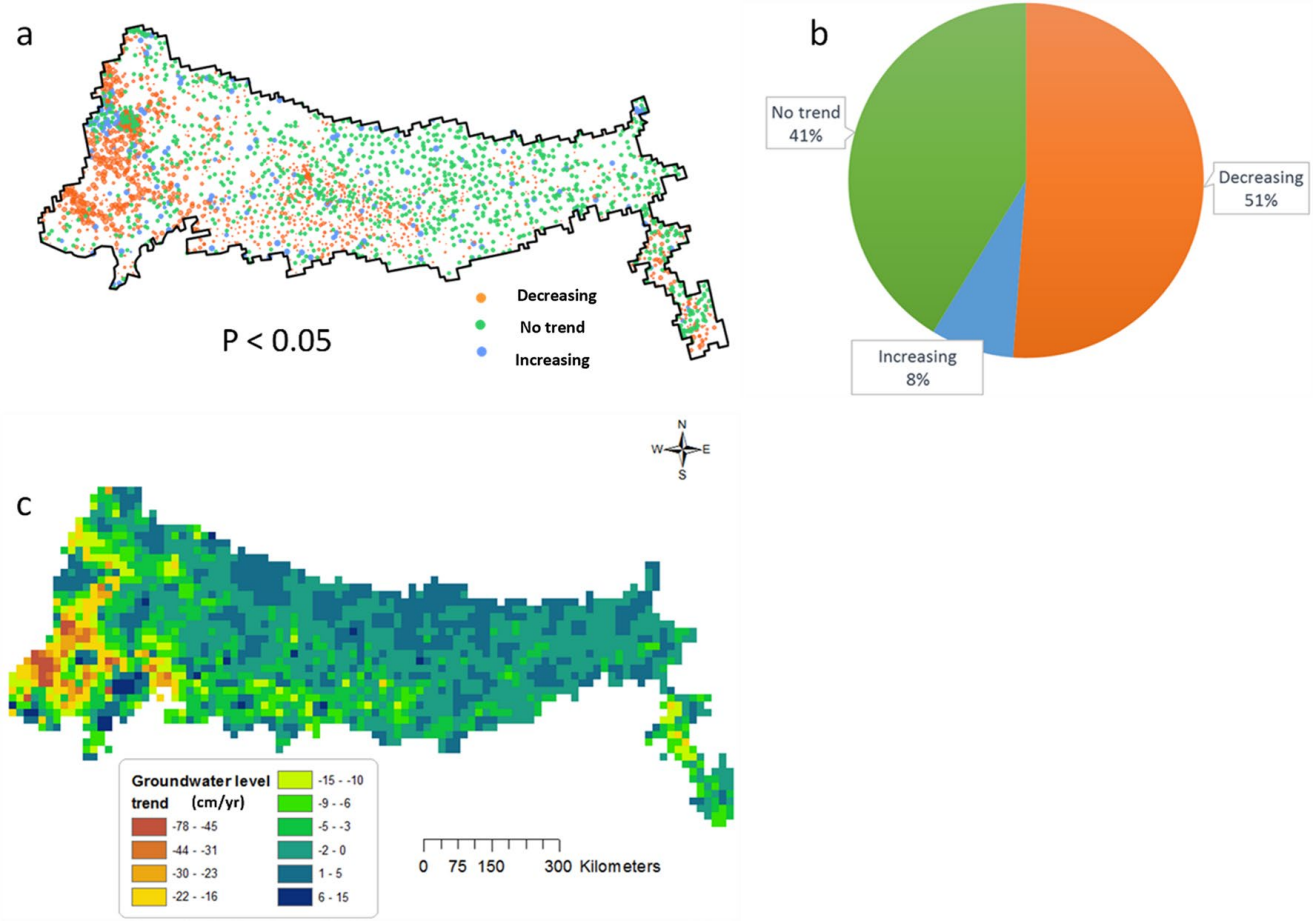
(**a**) Distribution of observation bores within the study area and trend obtained from the Sen’s slope. (**b**) Percentage of bores with increasing, decreasing and no trends. (**c**) Groundwater level trend

Figure 3a shows the locations of the 2851 bores in the Ganga Basin colour coded into three groups corresponding to groundwater level decreasing trends (orange), increasing trends (blue) and no significant trends (green). Fifty one percent of the analysed bores showed statistically significant decreasing trends in groundwater level (Fig. 3b). Eight percent (8%) of the bores showed statistically significant increasing trends in groundwater levels and remaining 41 percent of bores showed no significant trends indicative of steady groundwater levels.

Sen’s slope was calculated for all 2851 bores. Spatial plot of the Sen’s slope interpolated from these observations is shown in Fig. 3c. It may be observed from 3c that high rates of groundwater level decline are observed in the west and southwest areas. This includes agriculturally intensive areas in states like Haryana as well as urban areas like Delhi and Agra. The median value of the Sen’s slope obtained for the whole region is – 22 cm year^−1^ with a standard deviation of 121 cm year^−1^. Considering average specific yield of the aquifer as 0.12 (based on the modelling analyses), this equates to average decline in the groundwater storage in the aquifer at a rate of − 2.6 cm year^−1^. This indicates that, on an average, groundwater level has been declining in this region between 1996 and 2017. The large standard deviation is indicative of the large spatial variability in groundwater level trend across the aquifer.

## GRACE-derived groundwater storage anomaly

**Figure 4:**
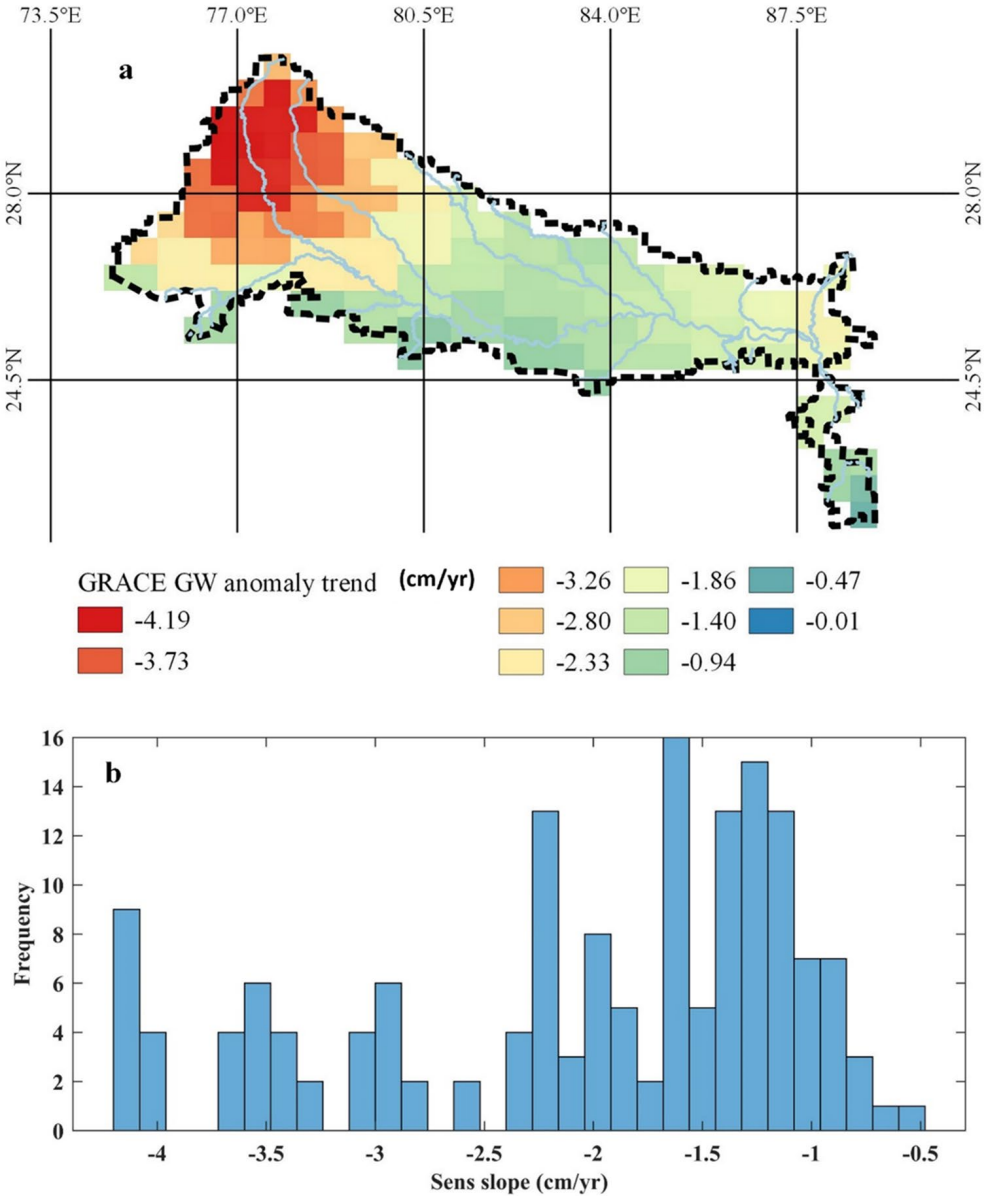
(**a**) GRACE groundwater storage anomaly trend (cm year^−1^). (**b**) Distribution of observed trends.

The anomaly in individual components of Total Storage Anomaly (T_sa_) are computed by removing its long-term monthly mean (2003–2016). The magnitude of the trend in GW and its significance is estimated using the seasonal Mann–Kendall test (seasonal M–K test) and the Sen Slope estimator. The trend from GRACE estimates (Fig. 4) follows a similar pattern as that observed from in-situ measurements shown in Fig. 3. The GRACE data analysis for the same region indicated storage anomaly in the range − 1.0 to − 4.2 cm year^−1^ with a median value of − 1.67 cm year^−1^ and standard deviation of 0.97 cm year^−1^.

## Groundwater modelling

Analysis of groundwater level data indicated a general declining trend in the study area. This warranted the investigation of groundwater dynamics and storage changes using a numerical groundwater model to understand storage trends across the region considering the hydraulic characteristics of the aquifer. This enables the exploration of uncertainties in the storage changes, which depend largely on hydraulic characteristics such as specific yield and hydraulic conductivity. Development of a probabilistic numerical model that accounted for the uncertainties in recharge and discharge components of water balance, as well as uncertainties in the hydrogeological characteristics enabled reliable estimation of changes in groundwater balance and its effect on long-term storage trend.

**Figure 5:**
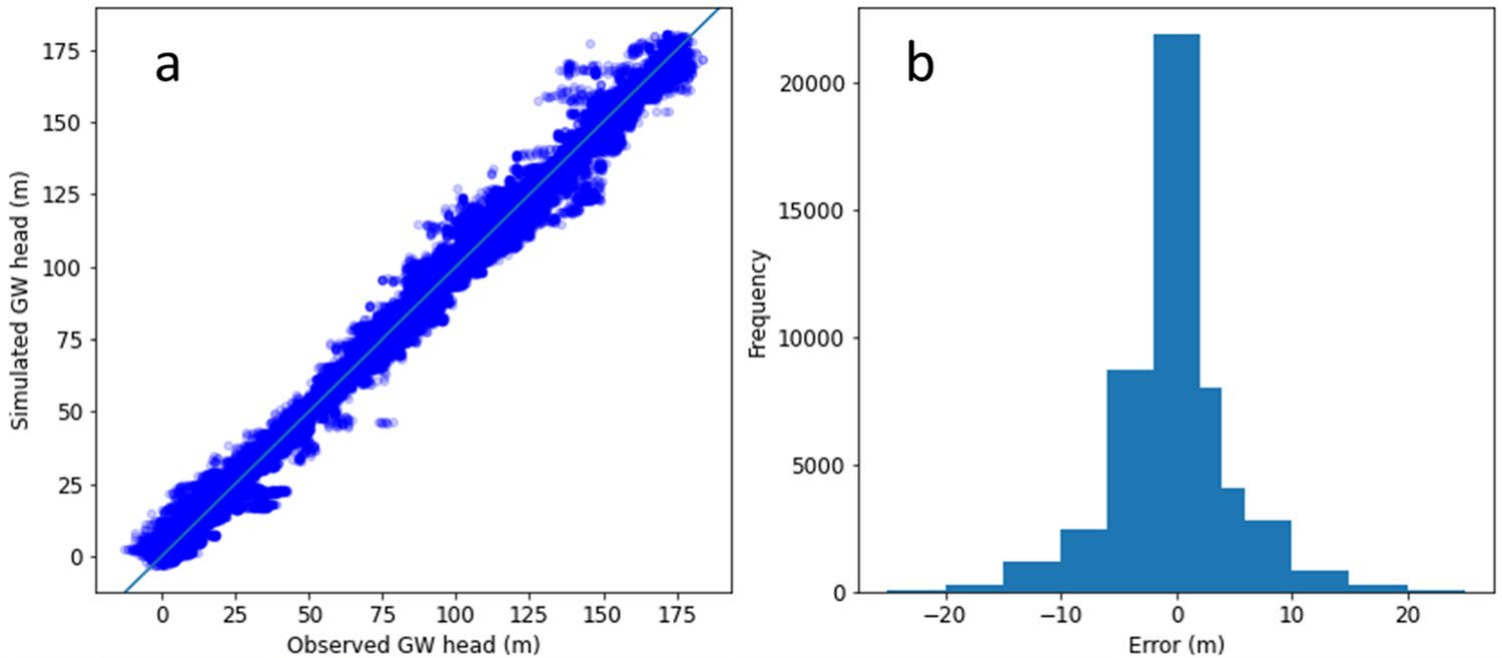
Match between simulated and observed groundwater head (**a**) and distribution of model calibration errors (**b**).

**Figure 6:**
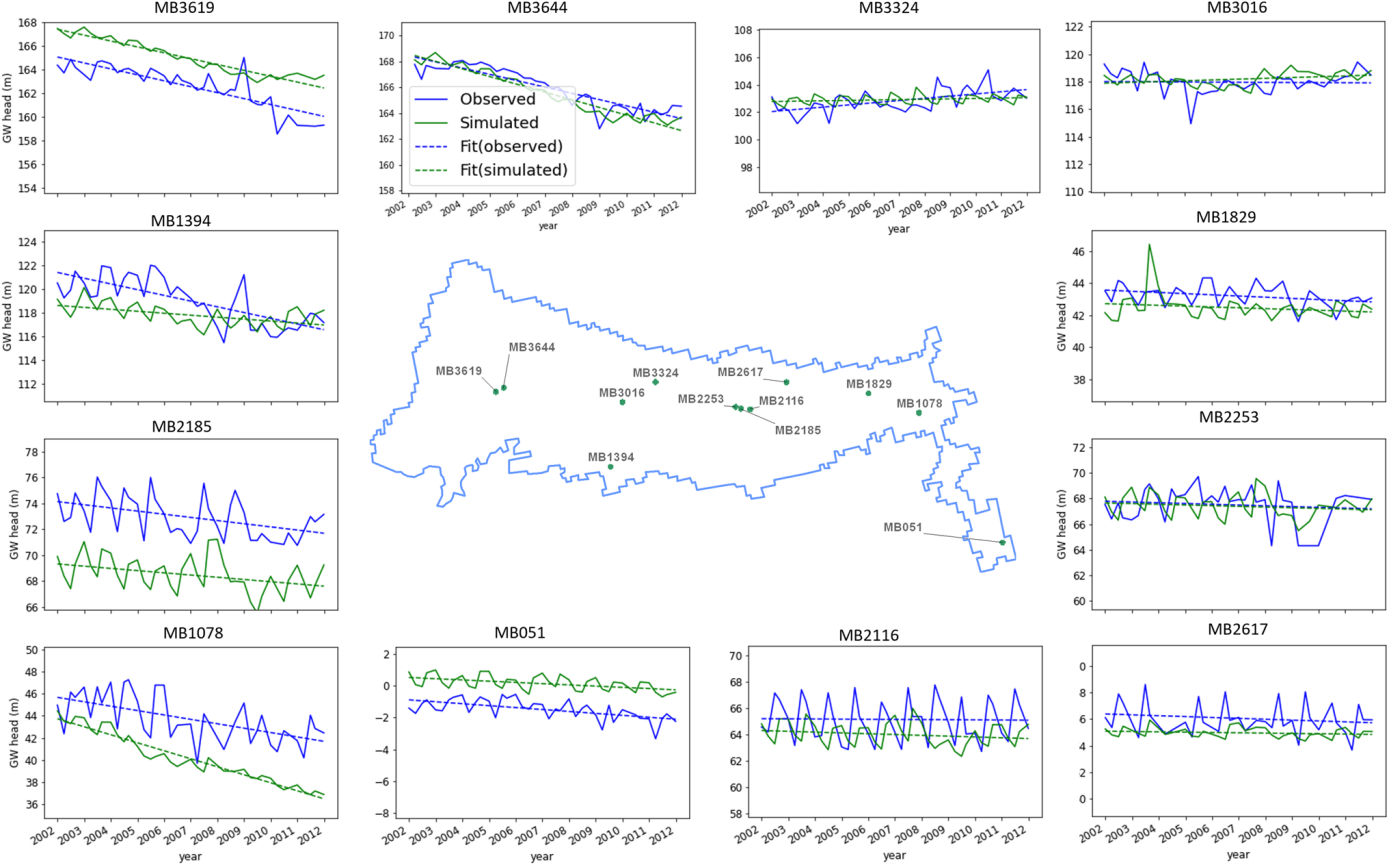
Observed and simulated groundwater heads at selected locations showing a wide range of trend types from steep declining (MB3619) to slightly increasing (MB3324) trends.

Calibration and uncertainty analysis of the numerical model using a probabilistic approach resulted in improved match between observed and simulated groundwater levels corresponding to the posterior distribution of model parameters (Figs. S1–S4). One realisation each of the river, recharge, hydraulic conductivity and specific yield parameters are shown in Fig. S5. The scatter plot of observed and simulated groundwater heads across all bores and corresponding distribution of calibration errors are shown in Fig. 5. Simulations use posterior parameter sets were able to capture the long-term declining trend (and increasing trend in smaller number of wells) in most wells. Examples of typical long-term trends in observed and simulated groundwater heads is show in Fig. 6.

**Figure 7:**
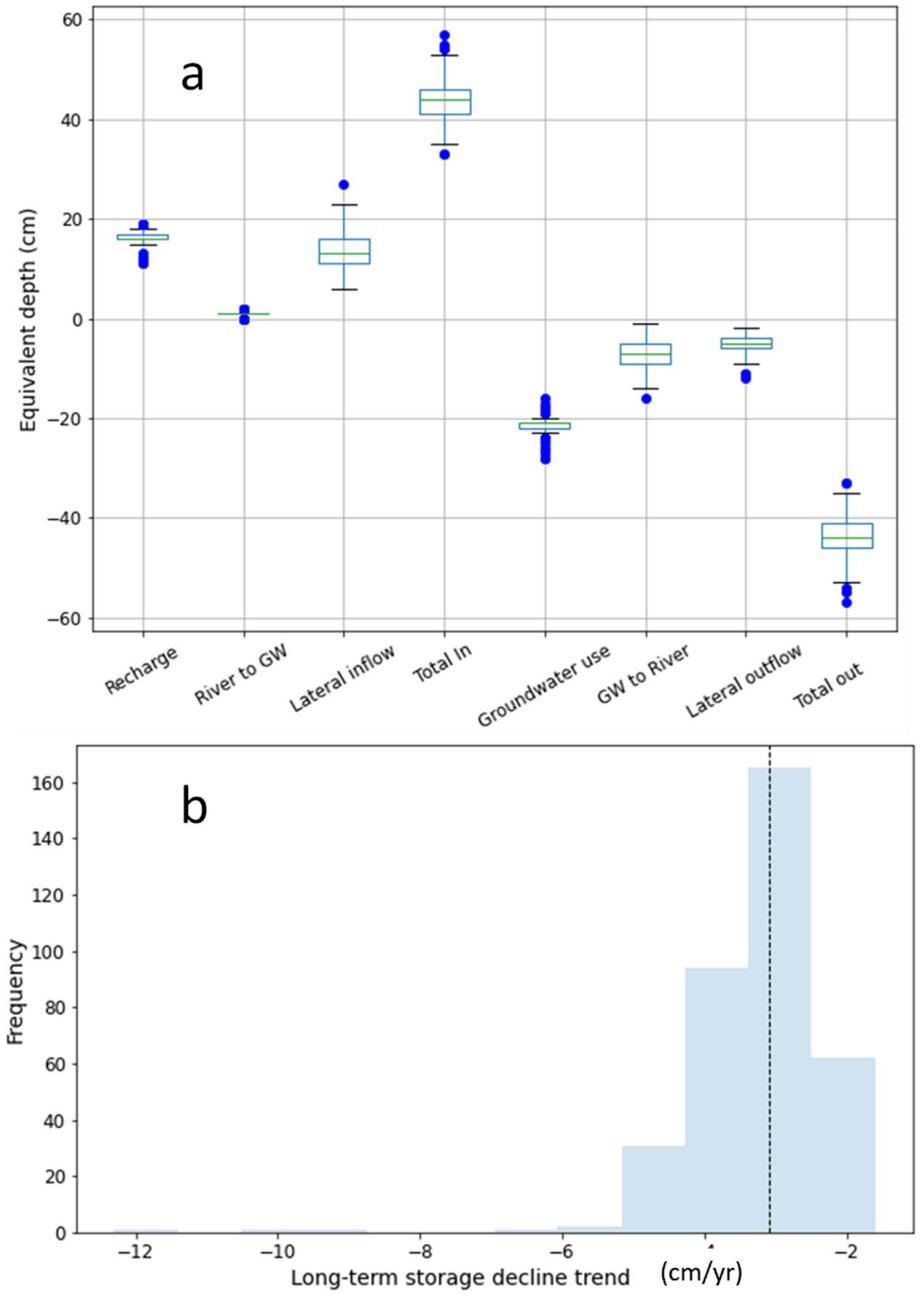
(**a**) Inflow and outflow components of probabilistic groundwater balance for the Ganga alluvial aquifer. (**b**) Distribution of estimated long-term average groundwater storage declining rate.

The probabilistic groundwater balance simulated for the regional alluvial aquifer is shown in Fig. 7. The major inflow into the aquifer is recharge from rainfall and irrigation excess with an estimated average value of 16.3 ± 1.2 cm year^−1^ and inflow from the lateral boundaries and river with estimated average values of 13.4 ± 3.6 cm year^−1^ and 0.8 ± 0.3 cm year^−1^ respectively. The major discharge component is the groundwater use (consumptive use) comprising pumping and groundwater contribution to evapotranspiration (ETg) with an estimated average value of 21.4 ± 1.3 cm year^−1^. Groundwater flow into river reaches is another major component of discharge with an average value of 7.0 ± 2.6 cm year^−1^. The volume of water entering and leaving the aquifer storage during each time step is calculated during the model simulation and represent the average groundwater flow that is stored in the aquifer and released out resulting in groundwater level changes. The difference between these two components over long period of time represents the rate of storage loss (or build up). The annual average rate of storage loss was computed as − 3.2 ± 1.0 cm year^−1^. This estimated value indicates that the average storage in the aquifer across the basin is declining.

**Figure 8:**
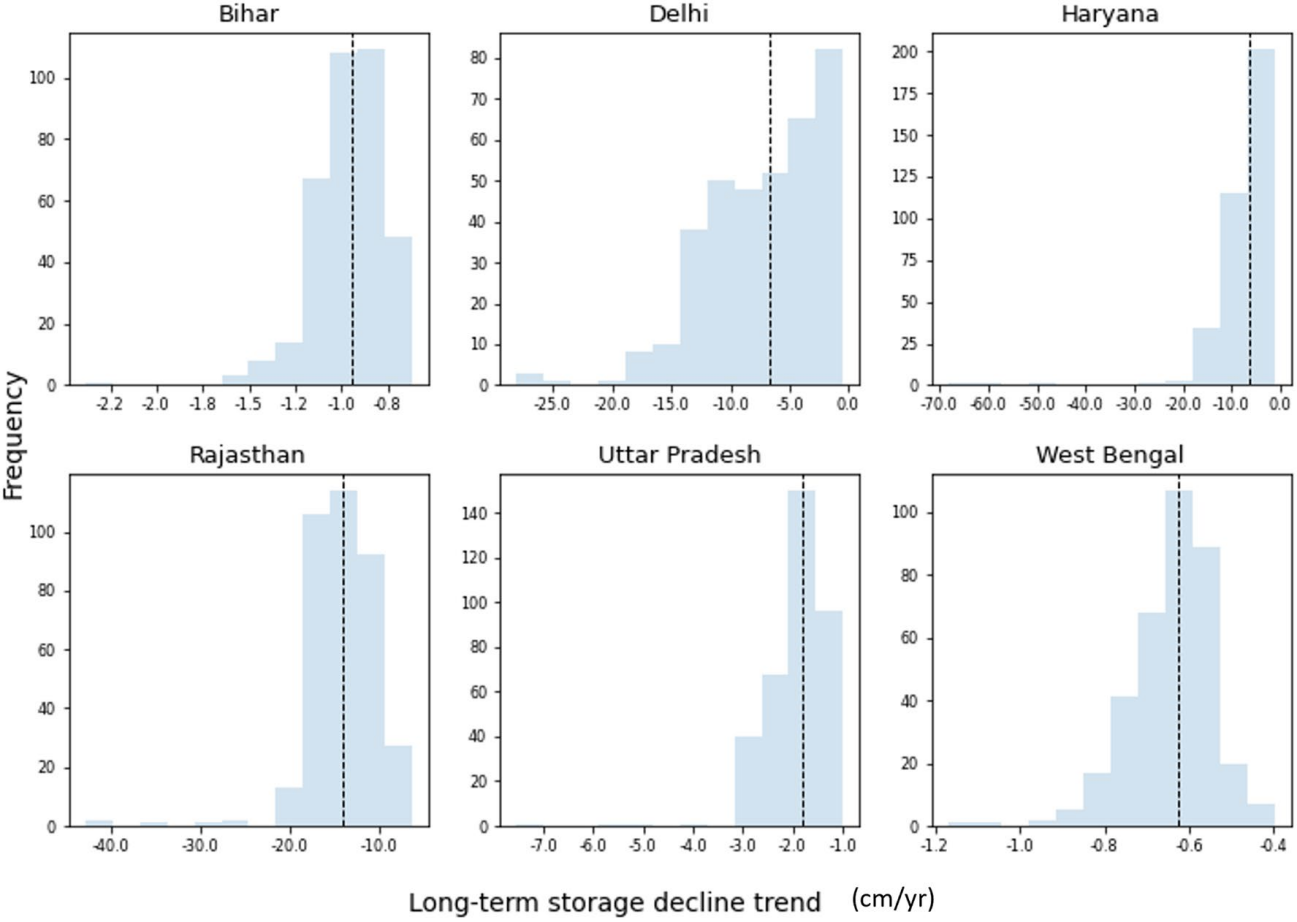
Distribution of simulated groundwater storage decline rate for 6 states located within the basin.

Groundwater balance and rate of storage decline was also analysed for the aquifer at the state level considering 6 major states within the basin. It was observed that groundwater storage loss is highest in the western states of the basin with the steepest average declining rate of − 14 ± 4.0 cm year^−1^ in the state of Rajasthan. The states of Haryana and Delhi have storage declines at − 7.5 ± 6.5 and − 7.2 ± 4.8 cm year^−1^ respectively. Uttar Pradesh, Bihar and West Bengal were estimated to have average decline in groundwater storage with rates of − 2.0 ± 0.7 cm year^−1^, − 1.0 ± 0.2 cm year^−1^ and − 0.6 ± 0.1 cm year^−1^ respectively. The distribution of simulated storage decline rates for these states are shown in Fig. 8.

**Figure 9:**
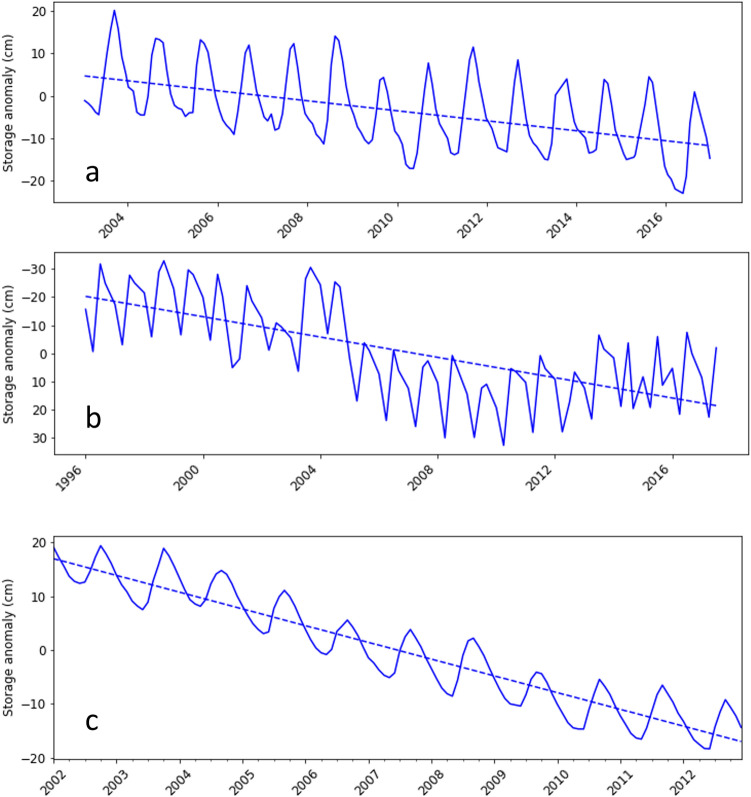
Trends in storage anomaly estimated from (**a**) GRACE derived groundwater storage anomaly (**b**) observed groundwater level depth to water table (**c**) simulated groundwater storage fluxes.

The trends in groundwater storage estimated from GRACE-derived groundwater storage anomaly, observed depth to water table and simulated groundwater storage fluxes are compared in Fig. 9. The trends are obtained by fitting linear regression model using Ordinary Least Square (OLS) implemented in the statsmodel python module^11^. Figure 9a shows the declining trend in storage obtained from the GRACE-derived storage anomaly. Similarly Fig. 9b shows the trend in storage calculated using observed depth to water table. Average of the observed depth to water table across all available monitoring bores was used to calculate the trend. Specific yield value of 0.12 was used to convert the anomaly in depth to water table to anomaly in groundwater storage. Figure 9c shows the declining trend in storage obtained using simulated groundwater fluxes obtained using the numerical model. Median of storage fluxes across 500 simulations were used for this purpose. It is noticeable that the direction and slope of the trendlines obtained using groundwater storage anomaly calculated the different approaches compare well. The slope indicates the rate of storage decline and the methods based on GRACE, observed water table and modelling respectively estimated it as − 1.7, − 2.6 and − 3.2 cm year^−1^ and corresponds well with the slopes observable in Fig. 9.

## Summary and conclusion

The three different approaches using different data sets that we used to compute long-term average groundwater storage changes in the Ganga Basin aquifer provided consistent results. The average groundwater storage loss was estimated as − 2.6 cm year^−1^, − 3.2 cm year^−1^ and − 1.7 cm year^−1^ respectively using the groundwater level trends, probabilistic groundwater modelling and GRACE storage anomaly approaches. The groundwater level trend indicated storage loss in the range − 1.1 to − 3.3 cm year^−1^ while the modelling and GRACE storage anomaly methods indicated the storage loss in the range of − 2.1 to − 4.5 cm year^−1^ and − 1.0 to − 4.2 cm year^−1^ respectively. The modelling analysis also indicated that the average groundwater storage is declining in all the major basin states, the highest declining trend being in the western states of Rajasthan, Haryana and Delhi. While smaller compared to the western states, average groundwater storage in states further towards east—Uttar Pradesh, Bihar and West Bengal within the basin are also declining. The probabilistic modelling framework developed in this study by integrating remote sensing and field data sets can be extended to identify districts with highest rates of storage losses and do scenario analysis pertaining to impacts from climate and other stresses and to inform basin planning and policy decisions for management.

## Methods

### Groundwater trend analysis

#### Mann Kendall Tau statistic

The time series of groundwater level observation between 1996 and 2017 was used for the Mann Kendall Tau and Theil Sen Slope estimation. The differences between pairs of observations in the time series was used for Mann Kendall Tau statistic computation. The ratio of all data pairs that increases or decreases with time are calculated separately. The difference between the two ratios represent the Tau statistic. Tau statistic values of 1.0 and − 1.0 represent monotonically increasing and decreasing trends respectively. Zero value of Tau statistic indicates equal number of increasing and decreasing pairs. Statistical significance of the trends calculated using the Tau statistic is indicated by the P-value. In this study a P-value of 0.05 was used as the cut-off for significance.

#### Theil Sen slope

While Mann Kendall Tau^12^ identifies statistically significant trends in observed groundwater levels. Theil Sen’s slope^13^ was used to quantify the rate of change of the levels. The method has been applied to calculate groundwater level trend^14–17^. Sen’s slope provides a robust estimate of slope of the trends identified using the Mann–Kendall Tau statistic. Sens’s slop represents the median slope among all lines fitted to all pairs of sample points in the time series^15^.

### GRACE storage anomaly

The Gravity Recovery and Climate Experiment (GRACE) satellite captures the time-variable gravity change induced by the mass redistribution within the atmosphere, ocean, hydrosphere and cryosphere from satellite gravimetry. This time-variable measurements are used to infer changes in terrestrial water mass by segregating it from other geophysical components^18^. GRACE observations are available in three different processed levels namely, Level1 satellite tracking data^19^, Level2 global gravitational stokes coefficients^20^, and Level3 global grids of change in terrestrial water storage (TWS)^21^. The latter two processed products provide information about the Terrestrial Water Storage (TWS) change which comprises of contribution from changes in canopy intercept, snow, soil moisture, surface water and groundwater storage. Previous studies^8,9^ have isolated groundwater information from GRACE TWS by estimating change in surface water component from the Global Land Data Assimilation System (GLDAS;^22,23^). The GRACE observations used in this study stems from the Mascon solutions processed by Center for Space Research (CSR-M)^24^.

In this study, groundwater storage anomaly was estimated from GRACE observations by segregating other sources of anomalies inducing geophysical components. As indicated in Eq. (1), the GRACE monthly TWS anomaly (TWS) measures the entire vertical water balance, which is made up of the SM anomaly (SM), snow water equivalent anomaly (SWE), surface water/reservoir storage anomaly (SW), canopy water content anomaly (CW), and groundwater anomaly (GW).

1 $${\text{TWS}}\_{\text{A }} = {\text{ SM }} + {\text{ SWE}} + {\text{ SW }} + {\text{ CW }} + {\text{ GW}}$$

2 $${\text{GW }} = {\text{ TWS }}{-} \, \left( {{\text{SM }} + {\text{ SWE }} + {\text{ SW }} + {\text{ CW}}} \right)$$

The GW measurement is derived from GRACE TWS data by removing other geophysical components that cause anomalies, such as SM, SWE, and CW. These components are obtained from a simulated Global Land Data Assimilation System (GLDAS;^22^) at 0.25° spatial resolution and 3 h intervals. On a monthly basis, they are transformed into the GRACE grid (0.5° × 0.5°).

### Probabilistic groundwater modelling

A single-layer numerical groundwater model for the Indian Ganga basin was developed using the MODFLOW code^25^ for probabilistic simulation of regional scale groundwater balance. A rectangular grid encompassing the study area was discretised into 322 rows and 665 columns resulting in square model cells with length of sides equal to 2.5 km. The model was set up with monthly stress periods to capture seasonal changes in model recharge and discharge components. The eastern and northern boundaries of the model corresponded to the political boundary of India with Bangladesh and Nepal respectively. These are not natural groundwater boundaries and hence head dependent flux boundary conditions were used to represent them in the model to simulate the predominant inflow of groundwater from the Terai plains of Nepal into the broader Ganga Basin in India as well as the predominant outflow of groundwater into to northwest region of Bangladesh. Model boundary on the west coincides with the boundary of the Ganga Basin. In the south the model boundary aligns with the extent of the alluvial aquifer.

### Model calibration and uncertainty analysis

A recently developed tool called Iterative Ensemble Smoother approach^26^ was used for model calibration and uncertainty analysis using the PEST +  + software suite. The PEST-IES offers an alternative approach for using the Jacobian matrix derived empirically from an ensemble of random parameter sets using the formulation proposed by Chen and Oliver^27^. Using this formulation, the model needs to be run only as many times as the size of the ensemble chosen thus eliminating the computational burden induced by the large number of parameters. This approach also enables the quantification of model parameter uncertainty. Since an ensemble of parameters are propagated through the algorithm until acceptable objective function values are attained, the calibration process ends up with an ensemble of model parameter sets that can all calibrate the model. This provides an estimate of the posterior parameter distribution constrained by the available observations^26^.

As described in the parameterisation section, pilot points were used to represent the spatial heterogeneity of hydraulic properties resulting in a total of 230 model parameters. Hydraulic characteristics—horizontal hydraulic conductivity and specific yield for the single model layer was represented by 186 pilot points. Eleven parameters were used to represent the scalers of recharge in 11 model zones. Similarly 11 parameters were used as scalers for evapotranspiration rate in the EVT package, another 11 parameters were used for informing the river conductance in these zones. Another set of 11 parameters were used for scaling the pumping rate. Including these zonal parameters for recharge, river, evapotranspiration and pumping there was a total of 230 model parameters. This implied that each iteration of the model calibration using the conventional algorithm would need the model to be run 230 times to populate the Jacobian Matrix. Model failure during computation of Jacobian matrix can also create problems.

The PEST-IES offers an alternative approach for using the Jacobian matrix derived empirically from an ensemble of random parameter sets using the formulation proposed by Chen and Oliver^27^. Using this formulation, the model needs to be run only as many times as the size of the ensemble chosen thus eliminating the computational burden induced by the large number of parameters. This approach also enables the quantification of model parameter uncertainty. Since an ensemble of parameters are propagated through the algorithm until acceptable objective function values are attained, the calibration process ends up with an ensemble of model parameter sets that can all calibrate the model. This provides an estimate of the posterior parameter distribution constrained by the available observations^26^. The details of representation of different recharge and discharge fluxes and corresponding parameters are described in the following.

#### Recharge

Land surface of the model area is subject to recharge from rainfall, overbank flooding, leakage from canal and irrigation excess recharge These were implemented in the model using the Recharge (RCH) package for MODFLOW to represent the major influx of water in to the groundwater system. Given the complexity of the recharge processes with several difference processes and components influencing it, a probabilistic approach was used to characterise recharge in the model. Diffuse recharge from rainfall is spatially varying with annual rainfall increasing from the west towards east in the region. Rainfall also varies temporally within the year, with higher amounts happening during the monsoon season. Rainfall recharge being the main component of recharge, it was assumed that spatial and temporal patterns in recharge is proportional to the rainfall. The model area was further divided into 11 zones based on DEM with distinct parameters for recharge assigned to these zones to accommodate aerial differences in recharge due to variation in rainfall patterns, presence of canal command areas, agricultural and irrigation patterns. Parameters were assigned to these zones whose values were constrained during the model calibration and uncertainty analysis step. Prior and posterior distribution of recharge obtained from the analyses is shown in Fig. S1.

#### River

The major rivers and selected tributaries were represented in the model using MODFLOW river package. Surface water flow data was not available for this study. Hence, SW-GW interaction was represented in the model using head-dependent flux boundary condition as represented by the river package. The water level variation was represented using interpolated data for the river boundary based on available secondary data sets including altimetry and in tandem with the topographic variation. The uncertainties induced by this and lack of measured river flows is explored by sampling the river conductance from a wide range and subsequently constraining it with available groundwater level observations. The river package facilitates exchange of water between river and groundwater. When the river stage is above the water table the river can leak into the groundwater. When the water table is above the river stage the groundwater model will contribute base flow in to the river. The exchange of water between groundwater and the river is governed by the hydraulic conductance of the river bed represented in the model. Zonal parameters were assigned for hydraulic conductance values for 11 zones of the model. The hydraulic conductance values were constrained during the model calibration process. Prior and posterior distribution of multiplication factors (river conductance factor) used forriver hydraulic conductancefor 11 zones and the average value across the basin is shown in Fig. S2.

#### Groundwater use

It is estimated that there are more than 10 million groundwater pumping wells extracting water from the alluvial aquifer of the Ganga Basin. The Central Groundwater Board (CGWB) has estimated values of groundwater abstraction for irrigation, industrial and domestic use at the district scale within the study area. Estimated district scale annual groundwater use for the study area was collated from CGWB (2019) and is shown in Fig. 2a.

While the district scale estimates of groundwater use was available, accurate information about spatial and temporal variability in groundwater use was not available for the region. Groundwater use for irrigation varies considerably between various seasons. Also, given the poor efficiency of irrigation in many areas, it is expected that a significant proportion of pumped water is returned to the water table as irrigation excess. Hence, the district-scale estimates were not hard-wired into the model as specified flux boundary conditions. Two different MODFLOW packages were used to represent the groundwater consumptive use. The evapotranspiration package was used to represent the consumptive use by vegetation and irrigated crops. This approach assumes that spatial and temporal patterns in irrigation water use is proportional to evapotranspiration estimates from remote sensing data set. Another component of groundwater consumptive use was represented using the MODFLOW well package. The groundwater pumping represented this way was assumed to be proportional to the district-scale estimates obtained from CGWB. The parameters pertaining to both these packages were adjusted during model calibration to match the historically observed groundwater levels. Thus spatially and temporally variable groundwater consumptive use was input into the model with parameters governing this rate for different zones in the model. Parameters pertaining to ET rate and ET extinction depth and groundwater pumping rate were considered as model parameters, the posterior distribution of which were obtained by constraining the model using observed groundwater levels. Prior and posterior distribution of multiplication factors for groundwater pumping for 11 zones and average value across the basin are shown in Fig. S3 and similar distributions of multiplication factors for the ET rate are shown in Fig. S4.

#### ET time series

ET time series data for constraining groundwater use was obtained from the Global Land Data Assimilation System (GLDAS). This estimate is obtained by assimilating satellite and ground based measurements in to the land surface models (LSM)^22^. The GLDAS estimates are available from different LSMs (such as Noah, VIC, CLM, Mosaic and Catchment land surface model) at different spatio-temporal resolution. For this study the monthly total actual evapotranspiration measured in millimetres of water loss is extracted from GLDAS for a period of 15 years from 2000 to 2015. The ET estimates are obtained from the Noah LSM, run at 0.25° spatial resolution. Spatial and temporal characteristics of groundwater contribution to ET was assumed to be proportional to these estimates.

### Model parameterisation

The model was parameterised in such a way that predictive uncertainty in groundwater balance owing to uncertainty in the model inputs and parameters could be explored during model calibration and uncertainty analysis. A zonation approach was used to parameterise the recharge, evapotranspiration and river package. The model area was divided into 11 zones based on topography. The topography generally slopes from west to east in the region. Thus, the zones align predominantly in the west to east direction which is also the predominant trend in rainfall intensity in the region. Each zone was assigned a multiplier for recharge which determines the percentage of spatially and temporally variable rainfall that reaches groundwater table as groundwater recharge.

Similar to one parameter was assigned to each zone which determines the percentage of total actual evapotranspiration that is contributed from groundwater. In addition to this, the extinction depth at which groundwater contribution to evapotranspiration ceases were also included as model parameter for each zone. Prior distribution of ET rate permitted the evapotranspiration rate to be assigned values that may be more than the total actual ET to simulate over irrigation practices that may be happening in many areas in the region. Representing irrigation water use by the evapotranspiration package also permits simulation of the conditions by which amount of irrigation water use decreases when water table falls below the suction limit and access by shallow wells become less feasible.

In this study river flow data was not available to constrain groundwater contributions to base flow and inflows from losing reaches during different seasons. Fluctuation in river level during different months was represented in the model by interpolated water level from available stage data including altimetry. Predictive uncertainties resulting from this is accounted in the model by including hydraulic conductance of the river bed as parameters for the 11 zones. The river inflows into groundwater and base flow into river is governed by these parameters. A wide range of values spanning seven orders of magnitude was assigned for these parameters acknowledging the large uncertainty that can exist for such parameters.

## Supplementary Information

Supplementary Figures.
