# Supplementary material for: Estimation of groundwater storage loss for the Indian Ganga Basin using multiple lines of evidence
Source: Sci Rep. 2023 Jan 31;13:1797. doi: 10.1038/s41598-023-28615-y (PMC9889759; doi:10.1038/s41598-023-28615-y)
Supplement: Supplementary file 1 — Supplementary Figures. [file 41598_2023_28615_MOESM1_ESM.pdf]

# Estimation of groundwater storage loss for the Indian Ganga Basin using multiple lines of evidence

Sreekanth Janardhanan[1], Akhilesh Nair[2], Indu J[3a,b], Dan Pagendam[4], Kaushika G S[5]

# Supplementary Information

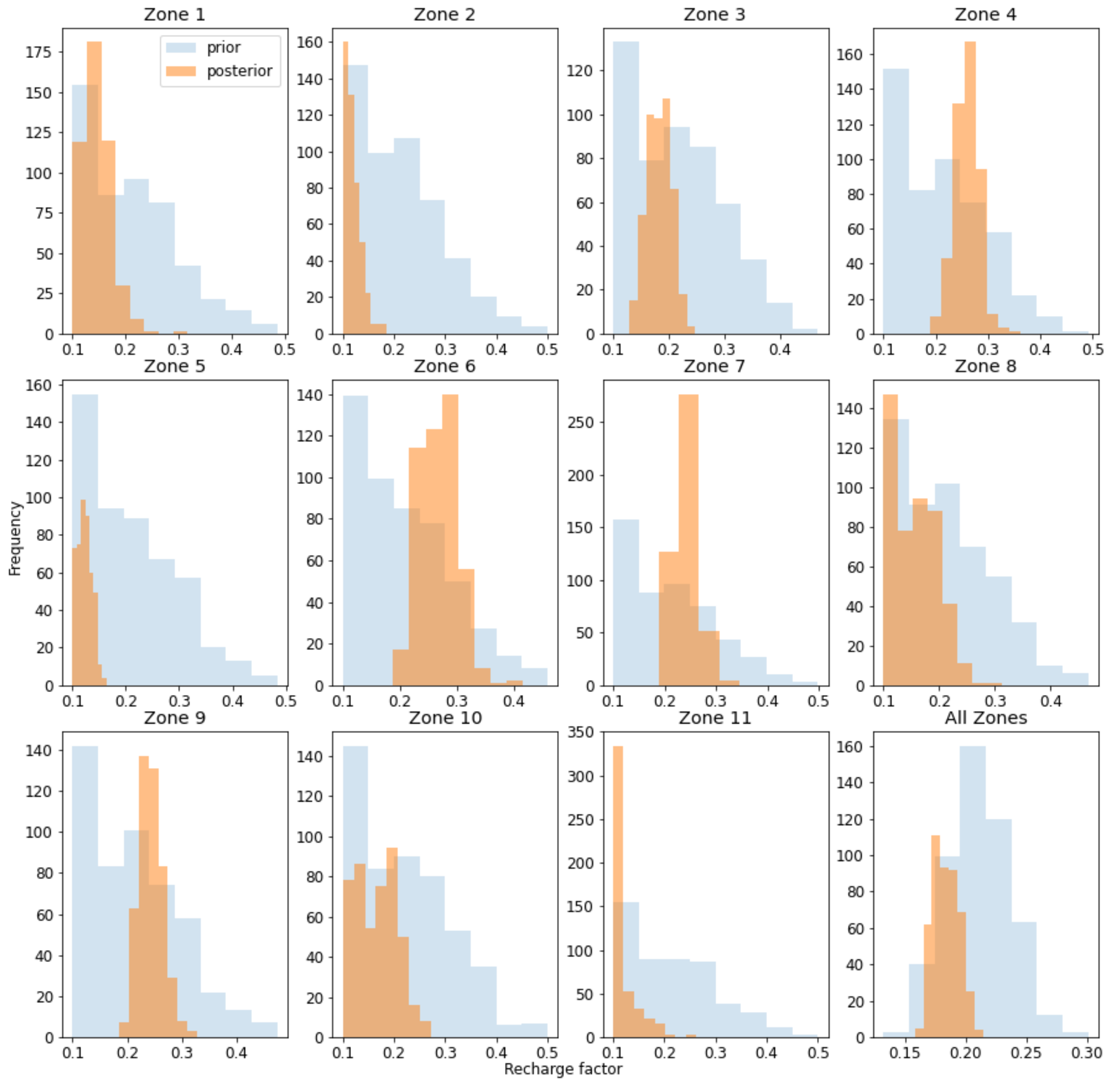


Figure S1: Prior and posterior distribution of recharge factor for 11 zones and the average for the whole basin

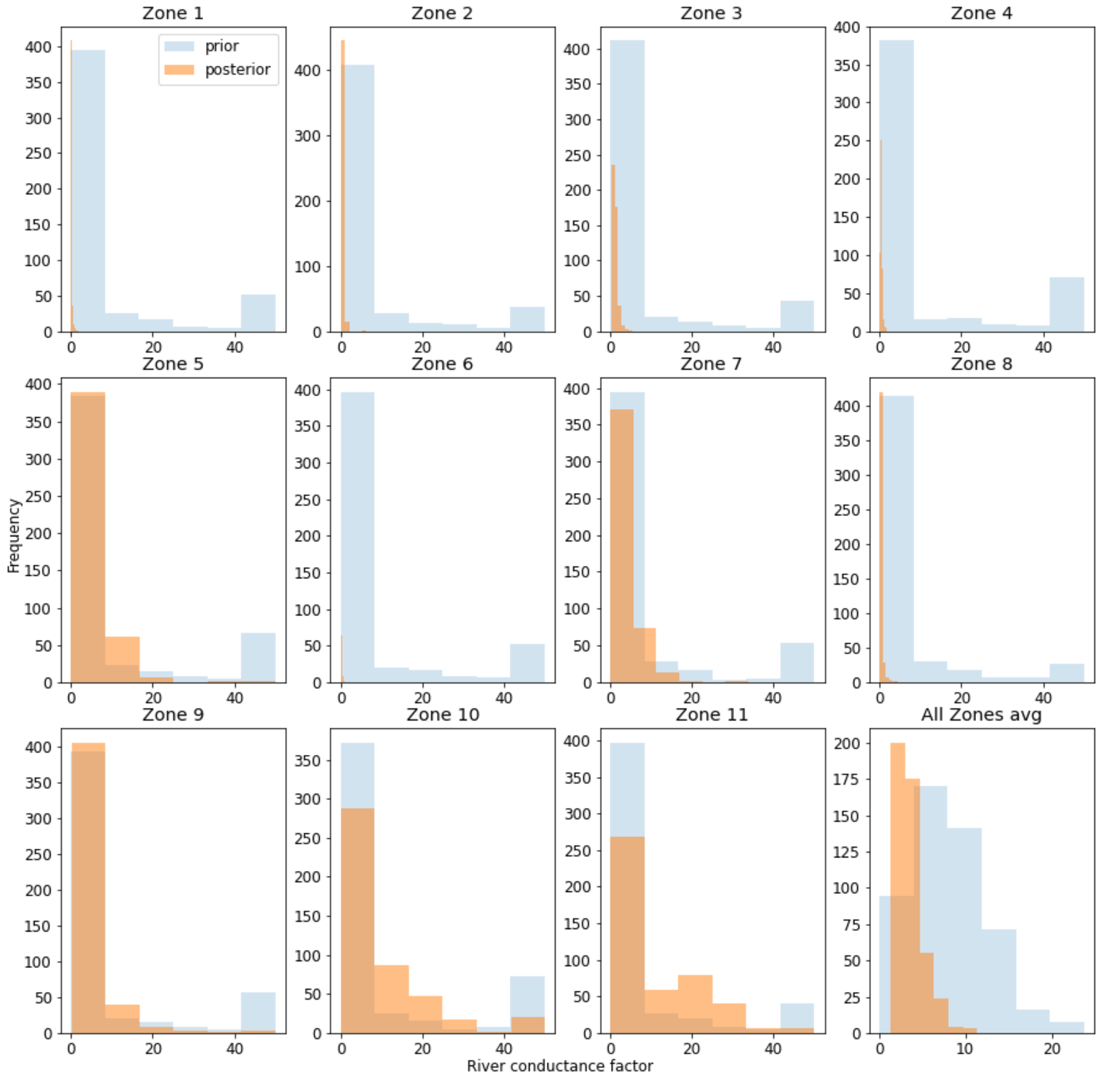


Figure S2: Prior and posterior distribution of river conductance factor for 11 zones and average for the whole basin

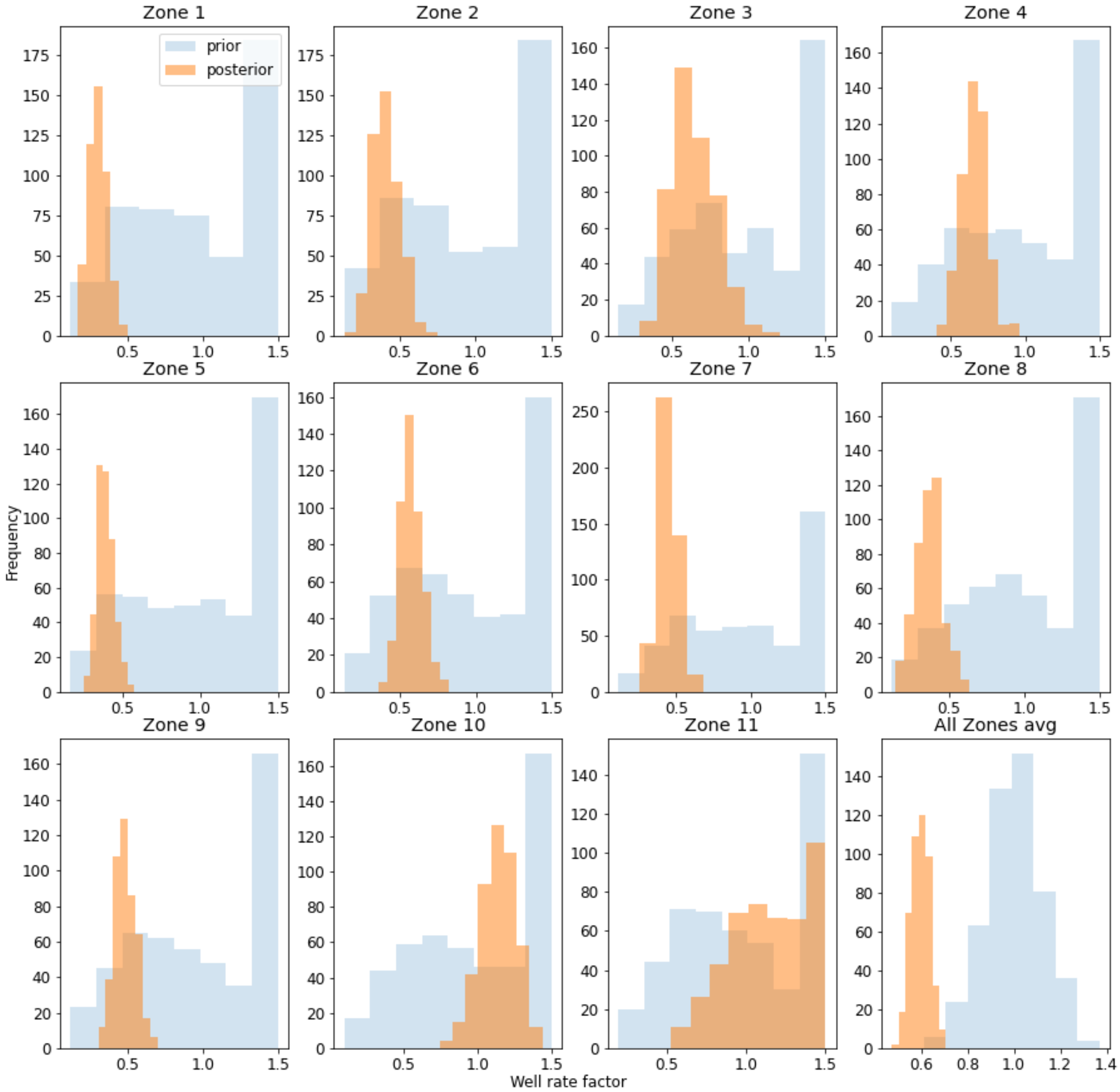


Figure S3: Prior and posterior distribution of groundwater pumping rate factor for 11 zones and average for the whole basin

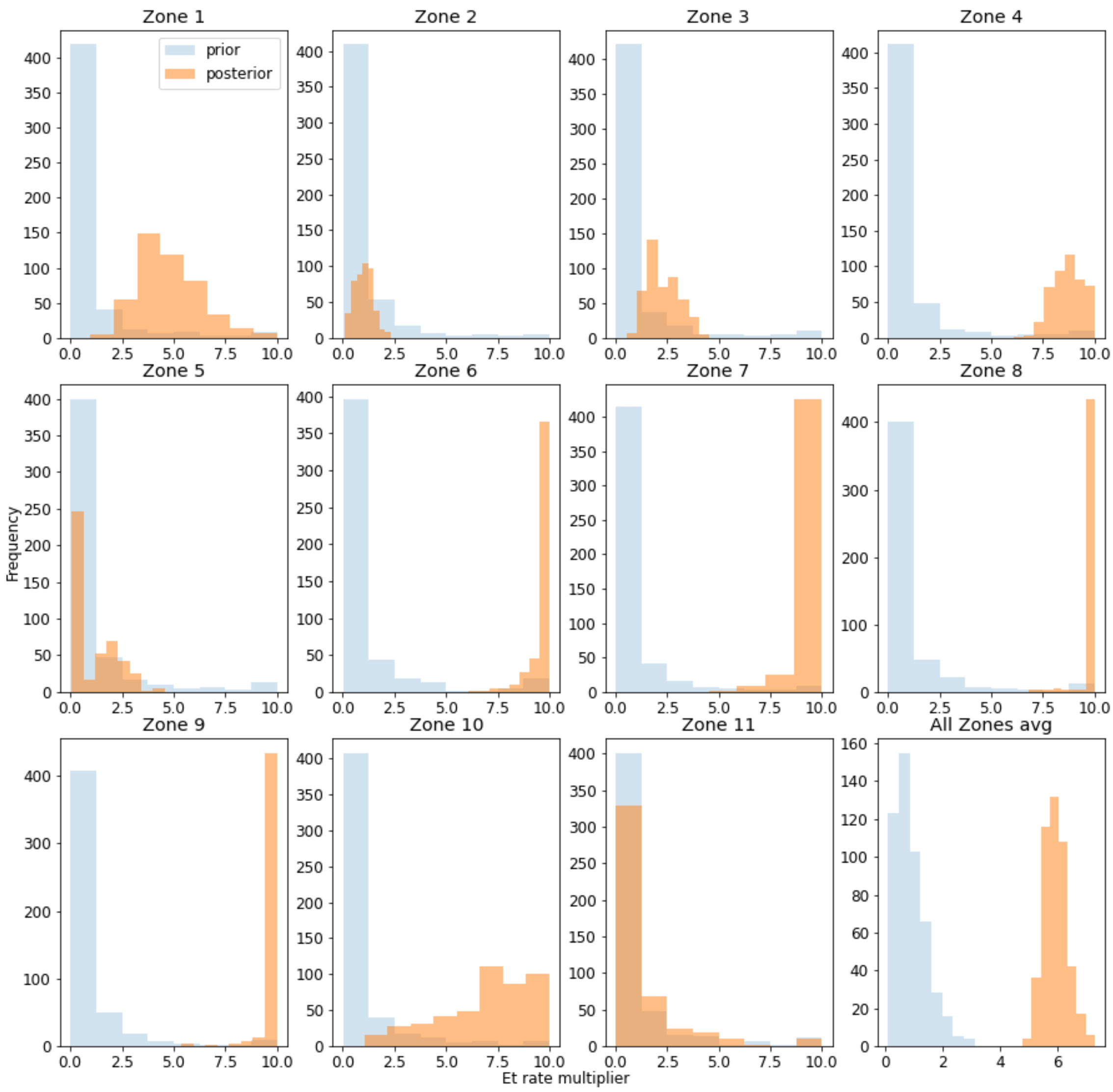


Figure S4: Prior and posterior distribution of groundwater contribution to actual ET rate factor for 11 zones and average for the whole basin

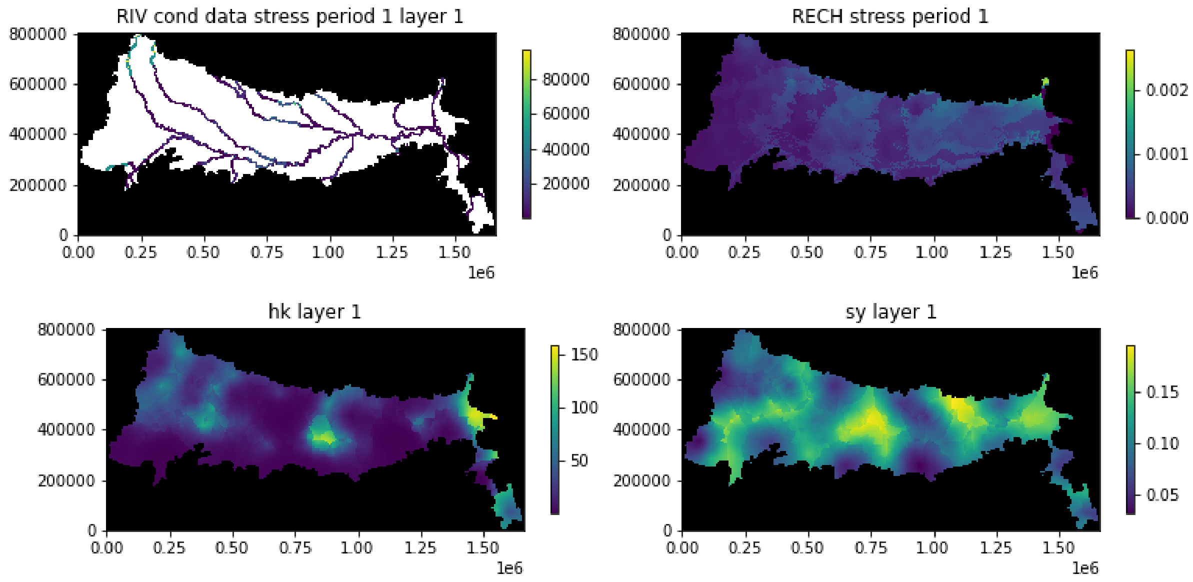


Figure S5: One realisation each of the river, recharge, hydraulic conductivity and specific yield parameters
